# The druggable genome: Twenty years later

**DOI:** 10.3389/fbinf.2022.958378

**Published:** 2022-09-30

**Authors:** Chris J. Radoux, Francesca Vianello, Jake McGreig, Nikita Desai, Anthony R. Bradley

**Affiliations:** Exscientia plc, Oxford, United Kingdom

**Keywords:** druggability, druggable genome, tractability, knowledge graph, drug target identification, artificial intelligence

## Abstract

The concept of the druggable genome has been with us for 20 years. During this time, researchers have developed several methods and resources to help assess a target’s druggability. In parallel, evidence for target-disease associations has been collated at scale by Open Targets. More recently, the Protein Data Bank in Europe (PDBe) have built a knowledge base matching per-residue annotations with available protein structure. While each resource is useful in isolation, we believe there is enormous potential in bringing all relevant data into a single knowledge graph, from gene-level to protein residue. Automation is vital for the processing and assessment of all available structures. We have developed scalable, automated workflows that provide hotspot-based druggability assessments for all available structures across large numbers of targets. Ultimately, we will run our method at a proteome scale, an ambition made more realistic by the arrival of AlphaFold 2. Bringing together annotations from the residue up to the gene level and building connections within the graph to represent pathways or protein-protein interactions will create complexity that mirrors the biological systems they represent. Such complexity is difficult for the human mind to utilise effectively, particularly at scale. We believe that graph-based AI methods will be able to expertly navigate such a knowledge graph, selecting the targets of the future.

## Introduction

Twenty years ago Hopkins and Groom (2002) published “The Druggable Genome.” This seminal paper recognised that only a subset of the newly published human genome (Lander et al., 2001) encodes proteins capable of binding orally bioavailable (Lipinski et al., 1997) molecules: the druggable genome. Over the last 20 years, many druggable genome variations have been published, focussing on either a specific disease area (Kumar et al., 2013), or including targets of biologics and more recent medicinal chemistry efforts (Russ and Lampel, 2005; Finan et al., 2017). Now, we believe that a data-rich knowledge graph of target-based annotations down to the level of individual residues will lead to the most complete description of drug target space—the landscape of all potential drug targets described by the many factors that determine a drug target’s quality. While this amount of data would be overwhelming for human user exploration, AI algorithms can expertly navigate these knowledge graphs to select the drug targets of the future.

Hopkins and Groom stated “druggable does not equal drug target”. Their original definition of “druggable” focused on proteins that can bind orally bioavailable drug-like molecules; however, this would now be thought of as drug-like ligandability. Contemporary definitions of druggability (Leach and Radoux, 2021) expand upon the original definition to include additional requirements, addressing the far more complicated question of “can this target yield a successful drug?” An ideal small-molecule drug target is disease modifying, capable of binding a selective, orally bioavailable molecule at a site that elicits a functional effect, has no on-target toxicity and is expressed in disease-relevant tissue. This multi-parameter problem is typically tackled by a multidisciplinary team, gathering information from literature, publicly available resources, and computational prediction on a per-target basis. First, we discuss what we believe is the state of the art and offer the next steps to provide the most complete description of target space to date. Second, we explore critical considerations for performing structure-based druggability at scale. We show how we are leveraging automation and cloud computing to expand our internal knowledge graph with residue-level annotations. Finally, we discuss how we think the arrival of AlphaFold 2 (AF2) will affect target assessment.

## Computer-readable annotation from gene to residue

Manually assessing all the factors that contribute to a suitable drug target is a time-consuming exercise. Fortunately, public resources such as Open Targets (Koscielny et al., 2017; Carvalho-Silva et al., 2018) and canSAR (Mitsopoulos et al., 2015; Mitsopoulos et al., 2020; Chau et al., 2016; Coker et al., 2018) bring together key data for target selection (Table 1). Open Targets focuses on linking targets to disease, but includes tractability data for small molecules, antibodies (Brown et al., 2018; Leach and Radoux, 2021) and PROTACs (proteolysis targeting chimeras) (Schneider et al., 2021). canSAR collates data from multiple sources and calculates structure-based, ligand-based, and network-based druggability scores, allowing users to assess the ligandability of specific cavities on each individual structure.

**TABLE 1:** Overview of data resources useful in the assessment of targets.

Resource	Focus	Data access
Open Targets	Target-disease association data, with tractability data for small molecules, antibodies, and PROTACs	User Interface
		JSON
		Parquet
		Apache Spark
		Google BigQuery
		GraphQL API
canSAR	Data and predictions for a range of areas applicable to drug discovery, including structure-based, ligand-based, and network-based druggability scores	User Interface
PDBe-KB	Functional annotations and predictions down to the protein residue level in the context of 3D structures	User Interface
		Neo4J Graph Database
		GraphQL API

These platforms provide function-rich user interfaces (UIs), which are powerful tools for scientists looking to discover future drug targets, but they do not allow exploration with AI approaches. Incorporating this data into a knowledge graph would allow additional data sources to be layered on top, allowing more complex queries and graph-based algorithms for data interrogation.

A good example of using knowledge graphs to annotate proteins with data is the PDBe Knowledge Base (PDBe-KB) (Consortium et al., 2021), which provides a neo4j graph database that maps data from several partner providers at the residue level. The growth of the PDB and improved protein structure prediction (Kryshtafovych et al., 2021) has increased opportunities for structure-based assessment. Additional considerations are necessary, however, to correctly identify therapeutically relevant pockets beyond simple ligandability prediction. Predicting whether pockets are orthosterically or allosterically functional, which offers opportunities for selectivity (Smilova et al., 2022), or are conserved across species (where required), provides key insights into a protein’s drug target suitability.

Structure-based druggability assessments typically focus on a single static protein structure (Hendlich et al., 1997; Hajduk et al., 2005; Cheng et al., 2007; Halgren, 2009; Huang, 2009; Kawabata, 2010; Volkamer et al., 2010; Volkamer et al., 2012a; Volkamer et al., 2012b; Krasowski et al., 2011; Desaphy et al., 2012; Borrel et al., 2015; Aggarwal et al., 2021), providing a score at the pocket level. Hotspot-based approaches, using either molecular dynamics (Young et al., 2007; Seco et al., 2009; Yang and Wang, 2010; Huang and Caflisch, 2011; Lexa and Carlson, 2011; Schmidtke et al., 2011; Bakan et al., 2012; Alvarez-Garcia and Barril, 2014; Ichihara et al., 2014; Vukovic et al., 2016; Arcon et al., 2017; Uehara and Tanaka, 2017; Vajda et al., 2018; Zariquiey et al., 2019; Yuan et al., 2020; Evans et al., 2021a) or static structures (Kozakov et al., 2015; Radoux et al., 2016; Curran et al., 2020), are capable of providing residue-level scoring. A hotspot-based assessment run at scale would provide residue level tractability annotations to be added to a knowledge graph such as the PDBe-KB, with all available structures for a given target used to calculate these scores.

In addition to structure-based assessment, drug discovery precedence for a target can be searched. This could mean identifying crystal structures in the PDB corresponding to drug-like compounds, or active drug-like compounds in ChEMBL (Mendez et al., 2018). The presence of multiple distinct chemical series further increases the chances that the target is tractable. If active compounds and protein crystal structures are not available, the target can be cross-referenced with published druggable genome sets (Hopkins and Groom, 2002; Russ and Lampel, 2005; Finan et al., 2017) to see if it is predicted to be tractable based on similarity to known drug targets.

Automation and scalability are essential in confidently expanding the druggable genome into novel and overlooked areas. Capturing all relevant data for target selection, from target-level evidence to per-residue data, in a single knowledge graph is a daunting but important task. Doing so will enable AI methods to undertake the work normally performed by large multidisciplinary teams and identify the very best novel targets.

## Structure-based druggability at scale

The human proteome comprises the protein sequences of all coding genes, including splice variants, from the human reference genome (Breuza et al., 2016). There are currently 20,360 human proteins in Swiss-Prot (Boutet et al., 2007), of which approximately 4,600 are implicated in disease according to the OMIM database (Hamosh et al., 2005), representing around 22% of human proteins with roles in disease. These proteins are the obvious subset of the human proteome likely to contain viable drug targets. An estimated 70% of the human proteome is covered by homologous protein structures (Somody et al., 2017), which can be exploited to characterise druggable pockets.

Where protein structures are available, they are often missing atoms, contain alternate atom placements, and are missing hydrogens. To obtain consistent high-quality structures, a method of automating the preparation of structures for computational experiments is required. Once a prepared set of structures is available, large-scale analysis requires a robust automation platform to locate target-binding sites across multiple structures per target.

It has long been known that considering proteins as rigid structures fails to consider energetic fluctuations that lead to proteins exploring a multitude of complex conformational states (Elber and Karplus, 1987). Moreover, important conformational changes in proteins are often associated with ligand binding; therefore, incorporating target flexibility into drug discovery pipelines will improve a project’s likelihood of success (Amaro et al., 2018). Molecular dynamics is one approach to incorporate protein flexibility; however, this is too computationally expensive to run at the proteome scale. Therefore, a rational approach to structure-based assessment would need to assess druggability across all structural data available rather than picking one representative structure. This is particularly important when data exist for multiple conformational states (e.g., active vs. inactive structures). Such an approach would necessarily yield a large amount of data and require careful analysis, especially in the context of automated pocket detection.

## Exscientia’s approach to structure-based assessment

**FIGURE 1:**
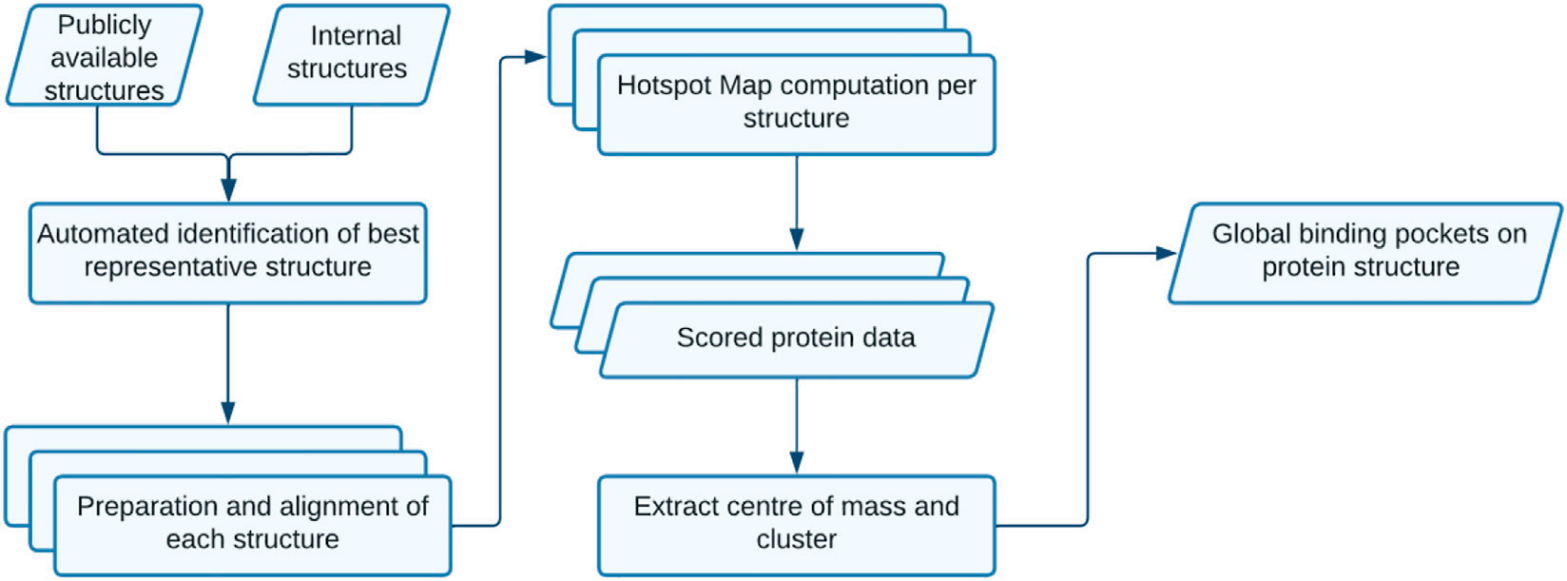
High-level schematic showing the overall structure-based assessment workflow.

Exscientia’s pipeline for automated target druggability assessments, summarised in Figure 1, has been designed to fulfil the above requirements. This pipeline captures a profile of druggability for each target that retains essential details such as single structures with non-conserved druggable binding pockets, while providing a global overview of the chosen target. Our workflows are run using scalable cloud computing infrastructure to facilitate the expansion of assessments to whole proteomes.

The first step leverages PDBe information relative to the structural coverage of each full-length protein characterised by a UniProt ID. The PDBe provides crucial information on the protein segments that are structurally enabled. Structural studies of larger proteins tend to yield multiple structures of shorter, non-overlapping segments. Our pipeline treats each of these segments separately, identifying the best representative structures available for each based on sequence coverage, resolution, and data quality.

For each individual target, publicly available structures are combined with in-house simulated and generated structures, and then aligned to the previously determined reference structure. All structural files undergo the same processing steps (related to rebuilding sidechains, protonating the protein and ligand, and assigning partial charges) to ensure data compatibility for downstream modelling tasks such as docking and molecular dynamics. The result of this step is a standard dataset of all structural data available for each target. Tractable binding pockets on each prepared structure are then assessed using Fragment Hotspot Maps with 3D grids, which highlight the areas most attractive to small molecules (Radoux et al., 2016; Curran et al., 2020). The centre of mass of each of these drug-like tractable volumes (which correspond to putative binding pockets on an individual structure) is extracted and stored.

**FIGURE 2:**
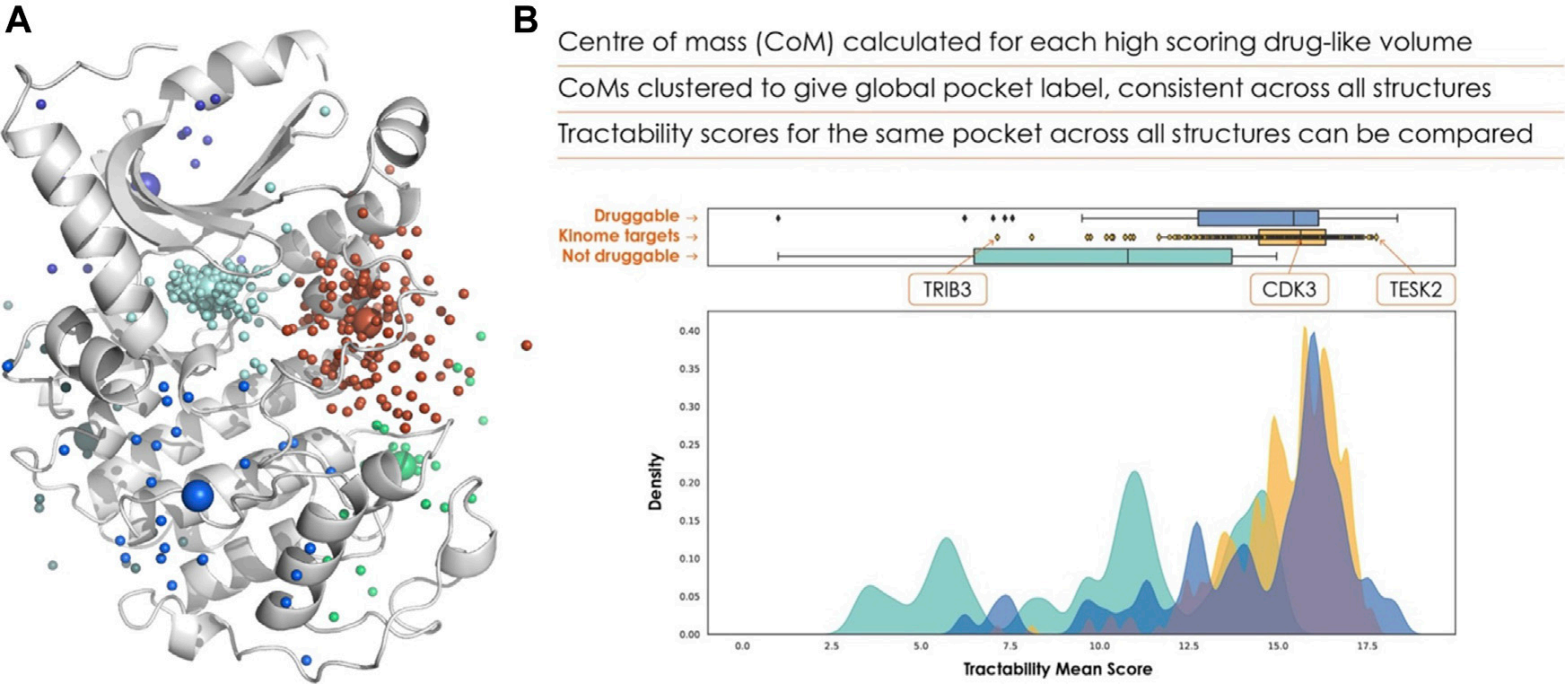
**(A)** Clustered pockets across all AlphaFold 2 structures of the human kinome, with the AlphaFold 2 model of cyclin-dependent kinase 7 (UniProt ID P50613). The cyan points show the ATP binding site of each kinase, allowing us to rank the kinome purely based on ATP-pocket druggability. **(B)** The frequency distribution of ATP site scores is shown in yellow, with known druggable (blue) and non-druggable (green) targets for comparison.

The centres of mass from all structures are clustered, shown in Figure 2, with each cluster taken to correspond to a distinct binding pocket, referred to as a global pocket. This is vital for referencing each binding pocket consistently across multiple structures, allowing tractability scores to be collated for each global pocket. This enables researchers to evaluate a range of scores for a given global pocket, and determine whether a target needs to adopt a particular conformation for effective binding.

## Assessing viral targets

With the ongoing COVID-19 pandemic, target selection for pandemic preparedness is of particular importance. The conservation of a drug target is assessed to capture the robustness of the protein and its binding pockets and used to provide valuable insights into the longevity of the drug in the face of resistance and pathogen emergence. These insights are obtained by computing a range of conservation metrics that rely on sequence alignments generated by MUSCLE (Madeira et al., 2019) for each target. The sequences that make up these alignments are identified using a BLAST (Altschul et al., 1990) search of the reference protein against a database of non-redundant protein sequences, restricted by taxonomies of interest. This enables a plethora of sequences of the target in the organism of interest to be captured, as well as orthologs for identifying drug activity against similar proteins.

At each residue in these alignments, we identify variants, the amino acid feature frequency across variants, and the most common amino acid frequency, which are mapped onto the reference PDB numbering. Additionally, we use Jensen-Shannon divergence (Capra and Singh, 2007) (JSD) to identify the conservation at each alignment position. JSD predicts functionally important residues using an estimator for sequence conservation. Each alignment position and its neighbour residues are compared to a background set of amino acids under no evolutionary pressure, and positions that differ substantially from this set are predicted as functionally important or constrained.

Functional insights from UniProt and FunPDBe are collated to identify amino acids that have interactions with small molecules or play a role in the target’s function. By extracting residue-level hotspot scores and mapping the conservation scores to them, we can quickly identify the most tractable and robust positions that can be used to inform design.

## Papain-like protease case study

**FIGURE 3:**
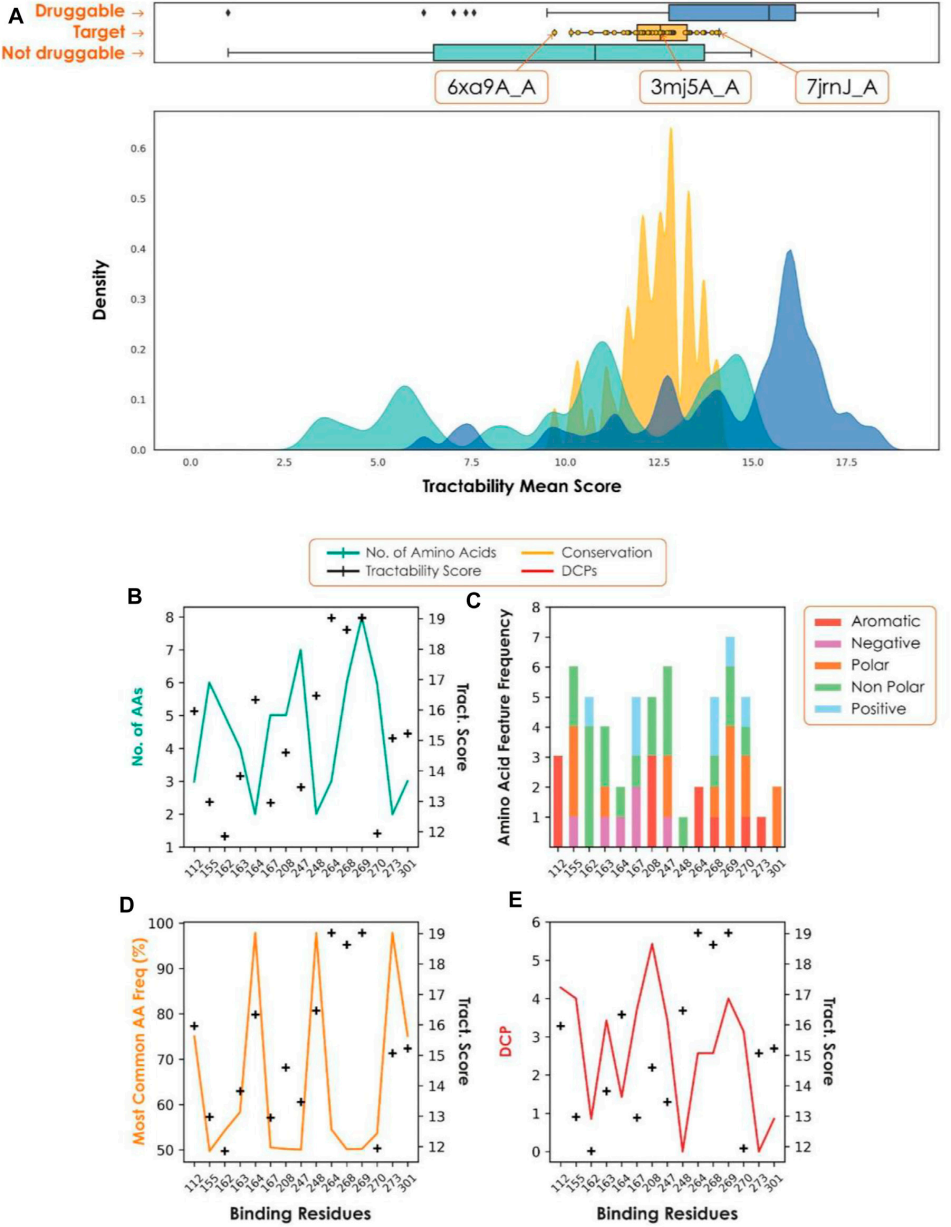
**(A)** Tractability for PLpro (yellow) vs. known druggable (blue) and non-druggable (green) targets. **(B**–**E)** Plots to show conservation metrics across coronavirus species HKU1, 229E, OC43, NL63, MERS, SARS-CoV-2 and SARS-CoV. In each plot, the hotspot tractability score for a given residue is shown as a black cross. **(B)** Number of different amino acids. **(C)** Number of amino acid properties. **(D)** Frequency of the most common amino acid observed. **(E)** Frequency with which an amino acid is differentially conserved between species for each predicted hotspot residue. A position is considered differentially conserved when it is conserved within a given species (JS-divergence score >0.8) and different from a corresponding conserved position in a different species. As we consider multiple species, this plot shows the average number of times a given position is labelled as differentially conserved.

Our recent assessment of the papain-like protease (PLpro), an attractive target for the treatment of COVID-19 (Shin et al., 2020), highlighted the importance of each step of the target assessment workflow. PLpro has a β-turn/loop formed by residues Gly266-Gly271 next to the active site (Osipiuk et al., 2021). Upon binding of a substrate or inhibitor, this loop closes, creating a more buried and druggable pocket. Assessment of only a single structure may have resulted in missing this difference, causing the PLpro site to be deemed not druggable. Consideration of all structures captures a range of druggability scores (Figure 3). Structures with a closed loop conformation are well within the druggable range, and the active site, Cys111, provides the additional opportunity for pursuing covalent strategies.

A fundamental requirement of viral target selection in the context of pandemic preparedness is that future variants and species will not develop drug resistance. Here we used conservation across multiple coronavirus species, combined with per-residue tractability scores, to assess the risk of drug resistance. In the case of PLpro, most of the residues with high tractability scores, and therefore essential for binding, were highly variable across a set of coronaviruses (SARS, MERS, 229E, NL63, HKU1, OC43). As a result, there is a high risk of future variants and novel coronaviruses being resistant to drugs developed against SARS-CoV-2 PLpro, making it unsuitable for pandemic preparedness.

## Scaling up to proteome-wide assessment

Our pipeline focuses on automating the process for single targets and runs using only a UniProt ID as input, which facilitates running the pipeline on a proteome-wide scale. Once this large-scale calculation is complete, and the results are captured in a knowledge graph, it can be layered together with public data from the PDBe and Open Targets, and our own internal knowledge graphs. Such a complete and data-rich description of the drug target space will enable far more precise search queries.

The more data included in the knowledge graph, the more complex these queries can become. We can consider further target annotations such as the subcellular location or tissue expression, or create edges between targets based on homology thresholds, pathway information or protein-protein interactions, mirroring the biological systems they represent. The benefits of introducing this structure and complexity can be exemplified by synthetic lethality, a promising area for novel cancer therapeutics. The first approved synthetic lethal therapy, for indications including breast and ovarian cancer, targets poly(ADP-ribose) polymerase (PARP). PARP is essential in cancer cells with BRCA1/2 mutations due to their defective homologous recombination (HR) pathway for DNA damage repair (Gutmanas et al., 2014). Healthy cells can survive PARP inhibition due to compensation by the HR pathway, allowing the BRCA-mutated cancer cells to be selectively killed. Using a knowledge graph as described above, pathways with similar function to the one containing an oncogene of interest, in this case DNA damage repair pathways for BRCA1/2 mutations, can be identified. These can then be searched for tractable functional binding sites to identify novel opportunities for synthetic lethality therapy targets. This could be done in a targeted way for a specific mutation or expanded to consider all identified oncogenic mutations and their respective pathways. Eventually, manually curated queries will likely become unwieldy, and graph-based algorithms will be the most effective approach for navigating this data-rich drug target space.

Many of the annotations do not rely on protein structure, but ultimately protein structures are vital for a pocket-centric target view. As well as helping the assessment of target tractability, a protein structure makes a project more doable by enabling structure-based design. Increased structural coverage of the proteome will have huge consequences on how much of the target space can be assessed and pursued.

## Impact of AlphaFold 2

The last 20 years have seen significant advances in the power and accuracy of homology modelling and *de novo* protein structure prediction methods (Pereira et al., 2021). These advances have recently culminated in the remarkable performance of AF2, developed by DeepMind, as demonstrated in the CASP14 experiment (Jumper et al., 2021a; Jumper et al., 2021b; Tunyasuvunakool et al., 2021). AF2 showed significant improvement from previous CASP experiments, with several structure predictions almost indistinguishable from experimental structures (Jumper et al., 2021a). Recently, more than 200 million AF2 structures have been released across more than 1 million species (Callaway 2022), greatly increasing the scope for structure-based assessment; however, care must be taken.

One of the most significant aspects of the AF2 method for structural bioinformatics and structure-based computational prediction has been the development of informative confidence metrics for local (pLDDT) and global (PAE) structural accuracy (Jumper et al., 2021a; Jumper et al., 2021b; Akdel et al., 2021). These metrics have been shown to estimate the local accuracy of AF2 predictions with remarkable reliability (Jumper et al., 2021a; Jumper et al., 2021b; Akdel et al., 2021; Pereira et al., 2021; Tunyasuvunakool et al., 2021). Specifically, at pLDDT scores >90 (high confidence), one estimation shows AF2 χ1 rotamers are 80% correct (Jumper et al., 2021b; Tunyasuvunakool et al., 2021). At pLDDT >70 (confident), AF2 has generally correct backbone predictions, although side chain conformations may be less accurate in these regions (Jumper et al., 2021b; Tunyasuvunakool et al., 2021). In addition, PAE scores may indicate domain orientation in multi-domain chains and possibly of proteins in multi-chain complexes (Akdel et al., 2021; Evans et al., 2021b; Tunyasuvunakool et al., 2021).

Since CASP14, DeepMind has released the AF2 code, model parameters and a database with AF2 model predictions (Akdel et al., 2021; Tunyasuvunakool et al., 2021). The AF2 database provides almost complete (98.5%) coverage of the human proteome, with structural predictions for all 20,000 proteins of the human proteome (Akdel et al., 2021; Tunyasuvunakool et al., 2021). Of the residues modelled, 36% were predicted with confidence (pLDDT >70), and another 22% were predicted with high confidence (pLDDT >90) (Akdel et al., 2021; Tunyasuvunakool et al., 2021). In terms of proteins, AF2 has confident predictions for >75% of protein sequence for 44% of human protein targets (Mullard, 2021). One analysis (Porta-Pardo et al., 2021) showed that the AF2 database increased the proportion of the human proteome with valuable structural insights from 47% to 75%, and reduced the number of proteins with no structural information from 4,832 to between 29 and 1,336 proteins (depending on confidence thresholds).

Having structural annotation and prediction for unannotated proteins and regions will benefit ligand binding-pocket predictions on those regions. Work by Beltrao (Akdel et al., 2021) indicates that while using low-confidence regions for binding-pocket detection can result in many false positives and negatives, predictions made on confident regions become comparable to using experimental crystal structures (Akdel et al., 2021). These preliminary results, however, will likely need further robust benchmarking, with more stringent filtering of homologs at both sequence and structure levels. Additionally, large-scale validation experiments will be required (Pak et al., 2021; Jones and Thornton, 2022).

While AF2 models can be good starting points for ligand-binding or pocket prediction, especially where there are few or no homologous structures available, it is essential to account for both confidence metrics and homology of proteins or regions of interest to the PDB (Akdel et al., 2021; Mullard, 2021; Tunyasuvunakool et al., 2021; Jones and Thornton, 2022). Understanding AF2 model limitations will be vital to ensure their impact on large-scale druggability predictions. With the correct preparation and consideration of confidence metrics, AF2 models will allow the predicted druggable genome to expand into areas previously not considered.

## Conclusion

In the 20 years since the publication of the druggable genome, tremendous advances have been made in multiple areas. These include improved structure prediction, structure-based assessment, public resources of collated data, and new architecture for data storage and methods for data interrogation. This provides the opportunity to build a detailed description of drug target space, an opportunity we must seize to select the very best future drug targets. The original druggable genome was represented as a simple Venn diagram, with “drug targets” at the intersection of “druggable genes” and “disease-modifying genes.” It is increasingly becoming a multi-dimensional problem, difficult to represent for human minds, particularly at large scales.

Graph-based AI algorithms can effectively work with data of this scale and complexity. Identifying the best method for selecting targets from a knowledge graph of this scale will be the subject of future research. Databases such as the Cambridge Structural Database (Groom et al., 2016) (CSD), ChEMBL (Mendez et al., 2018) and the PDB have all shown that bringing data together in an organised fashion allows far greater insights than from each individual data point.

## Data Availability

The original contributions presented in the study are included in the article’s supplementary materials, and any further inquiries can be directed to the corresponding author.
